# Comparing Real-Time Continuous Glucose Monitoring to Self-Monitoring of Blood Glucose: Advantages and Limitations for Children and Adolescents With Type 1 Diabetes

**DOI:** 10.7759/cureus.51496

**Published:** 2024-01-01

**Authors:** Reyof Aljuhani, Merna Adas, Raghad Alnaami, Reham Alshehri, Rahaf Alqarni, Sundus NoorSaeed, Abdulmoein Al-Agha

**Affiliations:** 1 Medicine, King Abdulaziz University Hospital, Jeddah, SAU; 2 Pediatrics, King Abdulaziz University, Jeddah, SAU; 3 Pediatrics, King Abdulaziz University Hospital, Jeddah, SAU

**Keywords:** hypoglycemia., glycemic control, t1d, smbg, rtcgm

## Abstract

Objectives

We aimed to determine the benefits and drawbacks of real-time continuous glucose monitoring (rtCGM) compared with those of self-monitoring of blood glucose (SMBG) in children and adolescents with type 1 diabetes (T1D) and their impact on glycemic control, hypo- and hyperglycemic episodes, exercise, quality of life, and psychosocial factors.

Methodology

This quantitative, comparative, cross-sectional study was conducted between July 2022 and March 2023 at the Pediatric Endocrine Outpatient Clinic, King Abdulaziz University Hospital, Saudi Arabia. Data were obtained via a clinical interview with children and adolescents with T1D aged 2-18 years.

Results

The study involved 121 participants, with 71 (58.7%) male patients and 50 (41.3%) female patients. The participants’ mean age was 11.9 ± 4.4 years. Compared with patients using SMBG, patients using rtCGM demonstrated a more significant decrease in the mean glycated hemoglobin (HbA1c) level from baseline (7.79 ± 1.17 vs. 8.92 ± 1.63, *P *= 0.001), a reduction in the number of hypoglycemic episodes (85.7% vs. 70.6%, *P *= 0.028), and stable blood glucose level during exercise (97.2% vs. 76.4%, *P *= 0.001). Additionally, 65 (92.9%) rtCGM users had undisturbed sleep compared with 22 (43.1%) SMBG users. Approximately 64 (91.4%) rtCGM users reported that the sensor helped decrease their anxiety levels and pain sensations.

Conclusions

In this novel study in Saudi Arabia, rtCGM demonstrated a significantly better impact than SMBG on glycemic control, hypo- and hyperglycemic episodes, and psychosocial factors in children and adolescents with T1D.

## Introduction

Diabetes is a chronic metabolic disease characterized by increased blood glucose levels, which result in severe organ damage. Access to affordable healthcare and treatment, including insulin use, is crucial for the survival of individuals with type 1 diabetes (T1D). Glucose monitoring in children and adolescents with T1D requires the use of a real-time continuous glucose monitoring (rtCGM) device or self-monitoring of blood glucose (SMBG) levels. Compared with SMBG, rtCGM could improve glycemic control as well as reduce its complications, length of hospital stay, and frequency of emergency room visits in individuals with T1D who are treated with insulin [1-4]. The preferred option for individuals requiring glucose monitoring is rtCGM, a subcutaneous sensor for measuring glucose levels in the interstitial fluid [5]. rtCGM provides a more detailed and complete assessment of the glucose profile, subsequently allowing for treatment adjustment [6]. The SMBG device is used for measuring the blood glucose levels in the capillaries, thus providing more accurate measurements [7]. However, measurement data are available only a few times each day, thus delaying therapeutic interventions; asymptomatic hypoglycemia may not be detected with a glucometer [6]. The rtCGM use improves glycemic control and reduces hypoglycemic episodes, regardless of the type of insulin therapy employed (multiple daily insulin injections or insulin pump therapy) [1,7,8]. We aimed to determine the benefits and drawbacks of rtCGM compared with those of SMBG among children and adolescents with T1D and their impact on glycemic control, hypo- and hyperglycemic episodes, exercise, quality of life, and psychosocial factors.

## Materials and methods

Study design

From July 2022 to March 2023, a quantitative, cross-sectional study was approved by the Biomedical Ethics Committee of King Abdulaziz University (HA-02-J-008), and verbal informed consent was obtained from the patient's caregivers before conducting clinical interviews. The study was held at the Pediatric Endocrine Clinic in the outpatient department of King Abdulaziz University Hospital, Jeddah, KSA. The study included 123 children and adolescents aged 2-18 years with T1D who used rtCGM of three different sensor models (Freestyle Libre, Abbott Diabetes Care Ltd., Oxon, UK; Dexcom sensor, San Diego, CA; or Medtronic sensor, Medtronic Plc, Minneapolis, MN) or SMBG for at least three months and had regular follow-ups within the past nine months.

Inclusion and exclusion criteria

Participants were included if they were aged 2-18 years, had T1D, and had undergone regular follow-ups (within three months) in the past nine months. Exclusion criteria include a diagnosis of type 2 diabetes, a diagnosis of T1D less than three months before the study, irregular follow-ups (missing appointments for more than six months) in the past nine months, skin allergies or disorders, hemoglobinopathies, or refusal to participate in the study.

Clinical interview questions

The in-sitting clinical interview conducted by the researchers and obtained from the caregivers included information on age, sex, nationality, socioeconomic status, diabetes duration, monitoring method, time in range (TIR), and HbA1c level in the last nine months. The rtCGM group had their estimated glycated hemoglobin (HbA1c) levels assessed. The interview questions were presented in Tables 1-2.

**Table 1:** Questions used to assess the rtCGM group. rtCGM, real-time continuous glucose monitoring; DKA, diabetic ketoacidosis; CGM, continuous glucose monitoring; HbA1c, glycated hemoglobin

Objectives	Questions
Glucose measurements	Does CGM help in reducing the number of hypo- or hyperglycemic episodes? Does CGM improve your HbA1c levels? Does CGM reduce the episodes of DKA/frequency of emergency visits?
Daytime management	Does CGM help with managing the blood glucose level during exercise (that is, swimming and football)? Does CGM help with the adjustment of pre-meal insulin doses? Does CGM help improve the quality of sleep? Does CGM help keep your diabetes under control on sick days? Does the presence of trend arrows help you manage hypoglycemia/hyperglycemia? Is the presence of trend arrows indicating hypo- and hyperglycemia a source of anxiety and concerns? Are CGM device alarms helpful (if available)? Does CGM help you relax, knowing that unwanted changes in blood glucose levels will be detected quickly? Does CGM help prevent problems rather than fix them after they occur? Does the presence of CGM on your child’s body bother you, him, or her?
Skin issues	Is the tape not strong enough and prone to peeling with increased sweating or swimming? Is CGM uncomfortable and does it cause too many skin-related issues (Will not stay on, rashes, itching, among others)? Is the management of CGM (hygiene rules, set change, among others) difficult and complex for you?
rtCGM use	Is the feedback from the device not easy to understand or useful? Did you have concerns about the accuracy and reliability of the sensor reading?
Cost	Is the CGM device too expensive to wear regularly?

**Table 2:** Questions used to assess the SMBG group. SBMG, self-monitoring of blood glucose; HbA1c, glycated hemoglobin

Objectives	Questions
Glucose measurements	Does SMBG help in reducing the number of hypo- and hyperglycemic episodes? Does SMBG improve your HbA1c level? Does SMBG reduce the episodes of DKA/frequency of emergency visits?
Daytime management	Does SMBG help manage the blood glucose level during exercise (swimming, football, or dancing)? Did the use of SMBG cause difficulties in performing exercises? Does SMBG help in the adjustment of pre-meal insulin doses? Does SMBG disturb your sleep? Does SMBG help keep your diabetes under control on sick days?
Skin issues	Is SMBG uncomfortable or painful to use? Have you noticed bruises on your finger since the time you used SMBG? Did you avoid testing your blood glucose level because of pain?
SMBG use	Is the interpretation of blood glucose readings not easy to understand? Did you forget to test your blood glucose frequently?
Cost	Is SMBG too expensive to use?

Definitions

Hypoglycemia was defined as a blood glucose level of less than 70 mg/dL (3.9 mmol/L) [9], whereas hyperglycemia was defined as a blood glucose level of more than 180 mg/dL (10 mmol/L) [10]. Average HbA1c values of three readings (three months between each reading) of less than 7.5% (58 mmol/mol) indicated good glycemic control, whereas values more than 9% (75 mmol/mol) indicated poor control [11]. Time in range (TIR) was the percentage of glucose readings between 70 and 180 mg/dL (3.9-10 mmol/L), with fasting glucose readings between 70 and 144 mg/dL (3.9-8 mmol/L) and postprandial glucose readings of less than 180 mg/dL (10.0 mmol/L) indicating good control [12,13]. Estimated HbA1c was the level obtained by the sensor compared with actual laboratory HbA1c results.

Statistical analyses

The collected data were coded, tabulated, and entered into IBM SPSS Statistics for Windows, Version 25 (IBM Corp., Armonk, NY) software. The Shapiro-Wilk test evaluated data distribution. Quantitative variables with normal distribution were expressed as mean and standard deviation, whereas skewed variables were expressed as median (interquartile range). Qualitative variables were expressed as frequencies and percentages. Student's *t*-test, Mann-Whitney *U*-test, and analysis of variance assessed statistical significance. The chi-square and Fisher's exact tests examined relationships between categorical variables. Pearson's or Spearman's method provided correlation analysis. Paired t-tests assessed differences between two mean values measured twice in the same study group. A *P*-value of less than 0.05 was considered statistically significant.

## Results

The study involved 121 participants with a mean age of 11.9 ± 4.4 years and a male-to-female ratio of 71 (58.7%) to 50 (41.3%). The mean age at diagnosis was 6.69 ± 3.83 years, and the mean disease duration was 5.16 ± 4.29 years. Regarding social class, 36 (51.4%) participants of the rtCGM group were from the high social class, whereas in the SMBG group, 9 (17.6%) belonged to the high social class. The majority (53, 75.7%) of the rtCGM group used the Freestyle Libre sensor, 13 (18.6%) used the Dexcom sensor, and 4 (5.7%) used the Medtronic sensor. In terms of glycemic control, there was a significant difference between the rtCGM and SMBG groups in terms of mean HbA1c levels (Table 3).

**Table 3:** Comparison between the two study groups in terms of HbA1c readings. HbA1c, glycated hemoglobin; SMBG, self-monitoring of blood glucose; CGM, continuous glucose monitoring

	Group				P
	SMBG		CGM		
	Mean	±SD	Mean	±SD	
HbA1c first reading	8.85	1.58	7.91	1.52	0.008*
HbA1c second reading	9.13	1.90	7.41	1.13	0.001*
HbA1c third reading	8.75	1.65	7.82	1.46	0.006*
Mean HbA1c reading	8.92	1.63	7.79	1.17	0.001*

**Figure 1:**
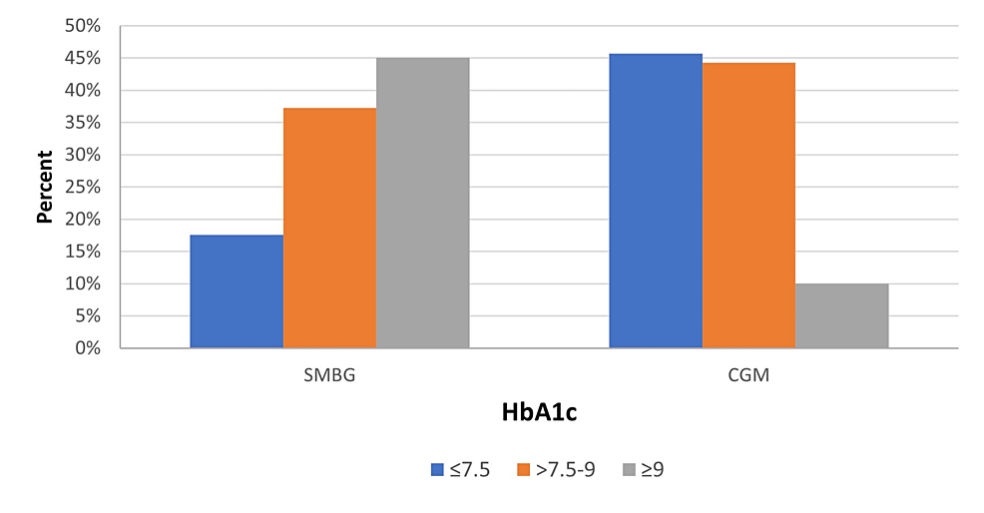
Difference between the rtCGM group and the SMBG group in terms of mean HbA1c levels. rtCGM, real-time continuous glucose monitoring; SMBG, self-monitoring of blood glucose; HbA1c, glycated hemoglobin

The two groups were further categorized into good, average, and poor control groups based on HbA1c levels. A highly significant difference was observed between the two groups in terms of mean HbA1c levels (*P *= 0.001), as shown in Figure 1.

However, in the rtCGM group, there was no significant difference between the mean HbA1c level in the last 6-12 months and the estimated HbA1c. Regarding TIR, a significant negative correlation was observed between the mean HbA1c level in the CGM group and TIR in the previous month and the last three months, but not in the first week. The clinical interview questions used for the rtCGM and SMBG groups, along with their advantages and disadvantages, are listed in Tables 4-5.

**Table 4:** Comparison between the rtCGM and SMBG groups in terms of advantages. rtCGM, real-time continuous glucose monitoring; SMBG, self-monitoring of blood glucose; DKA, diabetic ketoacidosis; SD, standard deviation

		Group				P
		SMBG		CGM		
		Mean	±SD	Mean	±SD	
Since usage, I noticed a reduction in the number of hypoglycemic episodes.	Disagree	15	29.4%	10	14.3%	0.028*
	Agree	18	35.3%	19	27.1%	
	Strongly agree	18	35.3%	41	58.6%	
Since usage, I noticed a decreased number of hyperglycemia.	Disagree	15	29.4%	18	25.7%	0.908*
	Agree	15	29.4%	22	31.4%	
	Strongly agree	21	41.2%	30	42.9%	
Since usage, I noticed an improvement in the average blood glucose level.	Disagree	16	31.4%	8	11.4%	0.025*
	Agree	11	21.6%	19	27.1%	
	Strongly agree	24	47.1%	43	61.4%	
Usage decreased the episodes of DKA/frequency of emergency visits.	Disagree	10	19.6%	17	24.3%	0.433*
	Agree	10	19.6%	8	11.4%	
	Strongly agree	31	60.8%	45	64.3%	
Usage helped keep my diabetes under control on sick days.	Disagree	8	15.7%	6	8.6%	0.149*
	Agree	12	23.5%	10	14.3%	
	Strongly agree	31	60.8%	54	77.1%	
Usage helped control the blood glucose levels during exercise	Disagree	12	23.5%	2	2.9%	0.001*
	Agree	12	23.5%	10	14.3%	
	Strongly agree	27	52.9%	58	82.9%	
Usage helped in the adjustment of premeal insulin doses.	Disagree	2	3.9%	3	4.3%	0.442**
	Agree	8	15.7%	6	8.6%	
	Strongly agree	41	80.4%	61	87.1%	

**Table 5:** Comparison between the rtCGM and SMBG groups in terms of disadvantages. rtCGM, real-time continuous glucose monitoring; SMBG, self-monitoring of blood glucose

		Group				P
		SMBG		CGM		
		n	%	n	%	
Too expensive to use	Disagree	21	41.2	17	24.3	0.102*
	Agree	8	15.7	10	14.3	
	Strongly agree	22	43.1	43	61.4	
The interpretation of blood glucose reading is not easy to understand.	Disagree	45	88.2	51	72.9	0.07**
	Agree	0	0.0	4	5.7	
	Strongly agree	6	11.8	15	21.4	

## Discussion

Glucose monitoring is fundamental to managing and preventing both short- and long-term complications of T1D. Consequently, pediatric endocrinologists have expressed an increasing interest in utilizing innovative technologies, like rtCGM, to accomplish this goal and decrease the risk of complications in children [14]. The rtCGM devices offer real-time, dynamic updates on the speed and direction of glucose levels, both high and low. The constant feedback from rtCGM can aid in well-informed management decisions that go beyond what is achievable with SMBG alone [15], potentially enhancing diabetes care for children and adolescents with T1D. Significant advancements in rtCGM technology have resulted in greater precision and convenience, including the 2016 US Food and Drug Administration approval of rtCGM use for diabetes management without requiring confirmatory blood glucose monitoring [16]. Fortunately, in Saudi Arabia, the government provides free access to rtCGM for all children and adults with diabetes, which is reflected in the fact that the majority of rtCGM users in our study were Saudi. The government provides the Freestyle Libre sensor model, which is the model that rtCGM patients most commonly use. The use of rtCGM has been associated with improved metabolic control in patients with T1D. A randomized clinical trial in the United States, including 153 T1D patients aged 14-24 years showed a 0.4% improvement in mean HbA1c level in the rtCGM group compared to the SMBG group over six months. The CGM group (mean HbA1c level: 8.4%) showed improvement compared with the SMBG group (mean HbA1c: 8.9%) [16]. In a six-month randomized clinical trial conducted in the United States, 154 T1D patients with baseline HbA1c levels ≥7.0% were enrolled and divided into three age groups (8-14, 15-24, and 25 years). HbA1c levels decreased in 47 patients aged 8-14 years (P = 0.85), and a higher CGM usage rate was associated with a more significant HbA1c decrease (*P* = 0.01) [17]. A meta-analysis of 16 studies, including 1,188 patients aged 15-43 years showed that rtCGM use was associated with a significant reduction in HbA1c levels compared with SMBG use (P = 0.002) [8]. In a study conducted in Turkey, 10 children aged 2-8 years were evaluated, and it was reported that using rtCGM devices for 9-34 months led to a significant improvement in HbA1c levels, with a mean HbA1c measurement of 6.81% [1]. In this study, the rtCGM users also demonstrated improvement in the mean HbA1c levels from a baseline of 3.7%, whereas SMBG users demonstrated improvement of 1.016% within nine months. The use of rtCGM is more advantageous than SMBG in recognizing hypoglycemic and hyperglycemic episodes, thereby promoting early intervention. A randomized controlled trial conducted in the United States involved 214 patients aged ≥8 years with T1D, and the study found that switching control patients who used SMBG to CGM reduced the rate of severe hypoglycemia by approximately 50% [17]. Another study in the United States recruited 55 parents of children aged 1-8 years diagnosed with T1D for at least six months, which reported that rtCGM was particularly useful in recognizing glucose excursions (hypo- and hyperglycemia) in children who could not describe or interpret their symptoms, especially in young children [18]. In this study, the frequency of hypoglycemic attacks in rtCGM users (60, 85.7%) was significantly lower than that in SMBG users (36, 70.6%; *P *= 0.028).

However, the frequency of hyperglycemic episodes was not significantly different between the two groups, with nearly similar frequencies of 70.6% (36) and 74.3% (52) in the SMBG and CGM groups, respectively. This finding could be explained by hypoglycemia phobia, which is common in the community, as well as complications other than hyperglycemia, which may make patients reluctant to undergo treatment to correct hyperglycemia. The rtCGM alarm feature is extremely helpful in managing hypoglycemia and hyperglycemia as it promotes early intervention. In a Turkish study conducted among 10 participants aged <9 years, alarms in the rtCGM system were found to help detect hypoglycemic and hyperglycemic episodes [1]. In this study, 52 (74.3%) participants reported that the rtCGM alarm helped them manage their blood glucose levels. Compared with SMBG, rtCGM allows patients to exercise more freely. A qualitative study in Turkey conducted among 10 children aged two to eight years found that rtCGM with remote monitoring enables children to play with friends without interruption [1]. In this study, 68 (97.2%) rtCGM users reported that this device helped manage their blood glucose levels during exercise, whereas 2 (2.9%) reported that the device was not helpful. Compared with SMBG users, 39 (76.4%) found it helpful in managing their blood glucose levels during exercise, whereas 12 (23.5%) did not find its use helpful (*P *= 0.001). The rtCGM device's trend arrows and alarms have provided users with reassurance regarding the early detection of any undesirable fluctuations in blood glucose levels, thereby alleviating their anxiety and concerns. A study conducted in Turkey involving 10 caregivers of children under the age of nine who used rtCGM for more than six months revealed that the trending arrows improved glucose control, reduced parental anxiety and concern, and enhanced the children's daily lives [1]. Additionally, study conducted in the United States involving 55 children aged between one and eight years found that the rtCGM's ability to remotely monitor blood glucose levels provided immediate access to the child's glucose level, leading to reduced parental concerns and increased confidence [18]. According to the latter study, 70 (100%0 participants reported that the trend arrows were crucial in reducing anxiety levels and concerns among rtCGM users and their parents by detecting changes in blood glucose levels promptly. Compared with SMBG, rtCGM with an alarm feature provides comfort and assurance during sleep. A US study with 222 participants showed that 66% of the respondents were awakened at least once a week by a hyperglycemia warning, and most patients benefited from the alarm by taking an extra correction bolus of insulin to improve glycemic control [19]. In a Turkish semistructured interview study involving 10 children aged two to eight years, rtCGM was reported to enhance blood glucose monitoring during sleep. This resulted in a reduced tendency to lose control at night and an improvement in sleep quality compared with SMBG [1]. This study also found that rtCGM uses improved sleep quality, with approximately 65 (92.9%) rtCGM users experiencing undisturbed sleep at night because of confidence that the device would only alarm if episodes of hypo- and hyperglycemia occurred during nighttime. Meanwhile, 29 (56.9%) SMBG users experienced interrupted sleep based on the need to wake up and check their blood glucose readings regularly. A systematic review conducted in Poland, which compared rtCGM and SMBG among 1,268 patients, confirmed that rtCGM systems were well accepted by patients with T1D and resulted in a reduced number of emergency room visits [4]. However, this recent study found no significant difference between the rtCGM and SMBG groups in terms of reduced episodes of diabetic ketoacidosis and frequency of emergency visits (45, 64.3% and 31, 60.8%, respectively) (*P *= 0.433). The trend arrows on the rtCGM device were found to significantly promote early detection of and intervention in hypo- and hyperglycemia. A semistructured qualitative interview study conducted in Turkey among 10 children aged two to eight years showed that the trend arrows helped parents detect hypo- and hyperglycemia trends early, determine the appropriate time for intervention, and select the proper dosage to avoid under- or overtreatment of their children [1]. According to a US survey of 222 patients with an average age of 46 years, rtCGM can help adjust premeal insulin doses and improve glycemic control. The majority of respondents changed their insulin timing based on the trend arrows, and many users showed a reduction in glycemic targets after using rtCGM [20]. In this study, the trend arrow was helpful for 67 (95.7%) rtCGM users and enabled the proper adjustment of insulin doses. In a qualitative study in Turkey, 10 children aged two to eight years and their families who used rtCGM reported that it reduced the number of confirmatory fingerstick testing to two to four per day and none on some days [1]. Another US study of 222 individuals with a mean age of 46 years found that 66% of participants confirmed the meaning of the rtCGM readings by performing SMBG when the sensor alarm alerted them about high glucose levels at night [19]. In this study, only 47 (67.1%) rtCGM users used SMBG to confirm rtCGM readings. Although rtCGM is a new technology, some patients may experience difficulties in using the device. An interview with 55 parents of children aged one to eight years who used the rtCGM device showed that users were overwhelmed by the frequency and amount of detail and found data interpretation challenging [19]. Messer et al. reported the same finding in a meta-analysis [20]. However, in this study, both study groups found it easy to understand and interpret the data obtained from the devices, with no significant difference between them (*P *= 0.07). Regarding the accuracy of rtCGM readings, a previous interview study of 10 children under the age of nine years found that some families were initially skeptical about the accuracy of the readings but gradually became more accepting. However, some families reported that the rtCGM device was less accurate outside the target range and displayed lower readings than the SMBG device during hypoglycemia, causing increased anxiety among family members [1]. A systematic review in Kansas City showed that rtCGM yielded more accurate readings when glucose levels were within the normal range [21]. Another meta-analysis of nine studies and 343 participants from different age groups in the United States showed that rtCGM does not always provide accurate data. However, many rtCGM users were not concerned about occasional inaccuracies, suggesting that trend data were more important and helpful than precision [20]. Approximately 43 (61.4%) participants in this study expressed concerns regarding the accuracy and reliability of the sensor readings. The monthly cost of using the rtCGM device is higher than that of using the SMBG device. While health insurance may occasionally cover some of the cost of rtCGM, families typically bear a significant out-of-pocket amount [21]. In a study by Karakuş et al. involving 10 children under the age of 9, it was demonstrated that rtCGM imposed a substantial financial burden on families. As they considered rtCGM essential, they had to make compromises to cover the cost [1]. Another US-based meta-analysis of nine studies and 343 participants from different age groups revealed that the cost of rtCGM is a considerable burden, with adults concerned about continuing its use in the future given the high financial burden and teenagers concerned about losing expensive devices [20]. In this survey, 20 (39.2%) SMBG users paid for the device out of pocket, with a median monthly cost of 300 riyals, while 31 (60.8%) received government assistance or insurance coverage. In contrast, 38 (54.3%) sensor users paid a median cost of 500 SR per month for the device, with the remaining 32 (45.7%) receiving government assistance or insurance coverage. In addition, hygiene rules and set changes for the rtCGM device were also considered. In a recent qualitative study in Turkey, families of 10 pediatric patients under the age of nine years were interviewed regarding their experiences with the cleanliness and set-change difficulties of using the rtCGM device. The families reported no significant issues during device removal, but some reported that using extra adhesives on their children caused pain during removal from increased depilation in the insertion area [1]. In this study, 52 (74.3%) rtCGM users reported no significant problems or difficulties in cleaning the device or changing its settings. A study by Karakuş et al. involving 10 caregivers found that maintaining the rtCGM sensor can be challenging. The caregivers used additional adhesives to protect the device from peeling from increased sweating and swimming, and 50% of the responders experienced skin irritation, itching, and sweating [1]. A meta-analysis of nine studies conducted by Messer et al. in the United States with 343 participants from various age groups reported that rtCGM users experienced increased pain and irritability from skin sensitivity, which resulted in discomfort, sensor rotation, and skin healing [20]. Approximately 48 (68.6%) participants in this study reported that the tape was insufficiently strong and peeled off when they sweated or swam, and 36 (51.4%) experienced discomfort or skin problems, while the remaining 34 (48.6%) did not. One of the main challenges of SMBG is lancing pain. However, a survey conducted in Italy involving 63 children and adolescents with T1D and their parents found that 80% of the participants reported that the placement of the glycemic sensor was less painful than numerous capillary injections using a conventional fingerstick, while the remaining 20% did not notice any improvement in painful symptoms [22]. In this study, 37 (72.5%) SMBG users reported experiencing discomfort or pain.

## Conclusions

This study conducted in Saudi Arabia demonstrated that both rtCGM and SMBG have benefits and drawbacks. However, compared with SMBG usage, rtCGM usage resulted in improved glycemic control and reduced hypoglycemic events, with benefits such as early detection of hypo- and hyperglycemic episodes; increased glycemic control at night, on sick days, and during exercise; improved sleep quality; and decreased anxiety in children and adolescents with T1D and their parents. Nevertheless, the main limitations of rtCGM include cost, interpretation accuracy, and tape adhesion issues, whereas SMBG was found to be painful and uncomfortable to use, and caused sleep disturbance. The study highlights the need for further education and research to address issues related to managing hyperglycemic episodes and improving TIR.*********
